# Crassicaudiasis in three geographically and chronologically distant Cuvier's beaked whales (*Ziphius cavirostris*) stranded off Brazil

**DOI:** 10.1016/j.ijppaw.2021.10.010

**Published:** 2021-11-06

**Authors:** Andrei M.B. Febronio, Gisele S. Boos, Renata L.G. Batista, Derek B. Amorim, Juliana P. Guimarães, Matheus V. Bianchi, Daniela B. Mariani, Letícia Koproski, Cristine Mari, Jociery.E.V. Parente, Luciana Sonne, Max R. Werneck, Sandra M.T. Marques, David Driemeier, Cristiane K.M. Kolesnikovas, Karina R. Groch, Caroline Sobotyk, Guilherme G. Verocai, Kátia R. Groch, Josué Díaz-Delgado

**Affiliations:** a Laboratório de Ecologia e Conservação - Centro de Estudos do Mar/UFPR, Universidade Federal do Paraná, Avenida Beira Mar, s/n, Pontal do Sul, 83255-000, Pontal do Paraná, Paraná, Brazil; b Setor de Patologia Veterinária, Faculdade de Veterinária, Universidade Federal do Rio Grande do Sul, UFRGS. Av. Bento Gonçalves - Agronomia, Porto Alegre, RS, 90650-002, Brazil; c Animal Service, Rua Vinte e Cinco, 49007, 134reia Branca, Aracaju, SE, Brazil; d Centro de Estudos Costeiros, Limnológicos e Marinhos (CECLIMAR), Av. Tramandaí, Campus Litoral Norte, UFRGS, 95625-000, Imbé, RS, Brazil; e Centro Universitário São Judas – Campus Unimonte, Rua Comendador Martins, 52, Vila Mathias, 11015-530, Santos, SP, Brazil; f Fundação Mamíferos Aquáticos, Estrada de Matapuã, 411, Anexo Chácara Anjo Gabriel, Mosqueiro, 49100-00, São Cristovão, SE, Brazil; g Instituto Brasileiro para a Medicina da Conservação – Tríade, Rua Silveira Lôbo, 52061-030, Recife, PE, Brazil; h Instituto BW para conservação e medicina da fauna marinha, Rua Professora Sueli Brasil Flores n.88, bairro da Praia seca, 28972-765, Araruama, RJ, Brazil; i Laboratório de Helmintologia Veterinária, Faculdade de Veterinária, UFRGS, Rua Vinte e Cinco, 91540-000, Porto Alegre, RS, Brazil; j Associação R3 Animal, Parque Estadual do Rio Vermelho, 88061-500, Florianópolis, SC, Brazil; k Instituto Australis, Av. Atlântica sn CP. 201 - Itapirubá Norte, 88780-000, Imbituba, SC, Brazil; l Department of Veterinary Pathobiology, College of Veterinary Medicine & Biomedical Sciences, Texas A&M University, 77843, College Station, TX, USA; m Texas A&M Veterinary Medical Diagnostic Laboratory, 483 Agronomy Rd., 77843, College Station, TX, USA

**Keywords:** *Crassicauda*, Cetacean, Bronchopneumonia, Marine mammal, Nematode, Parasitism

## Abstract

The Cuvier's beaked whale (CBW; *Ziphius cavirostris*) is a cosmopolitan marine mammal found in deep tropical and temperate waters of all oceans. CBW strandings have been recorded sporadically in Brazil; however, there is lack of information available regarding their causes of stranding and/or death. Herein, we report the epidemiologic, pathologic, morphologic parasitologic features and molecular identification of arterial and renal crassicaudiasis by *Crassicauda* sp. in three geographically and chronologically distant CBW stranded off Brazil. CBW-1 was an adult male stranded dead in Rio Grande do Sul State. CBW-2 was an adult female that stranded alive in Sergipe State and died shortly after. CBW-3 was and adult male that stranded dead in Santa Catarina State. The most relevant pathologic findings in these three CBW were severe, chronic proliferative mesenteric and caudal aortic endarteritis and chronic granulomatous and fibrosing interstitial nephritis with renicular atrophy and loss, and numerous intralesional *Crassicauda* sp. nematodes. Furthermore, CBW-1 had concomitant gram-negative bacterial pneumonia and pulmonary and hepatic thromboembolism. Morphologic analysis of renal adult nematodes identified *Crassicauda* sp. in the three CBW. Molecular analyses targeting the 18S and ITS-2 ribosomal loci of renal nematodes in CBW-2 and CBW-3 identified *C. anthonyi*. It is believed that severe arterial and renal crassicaudiasis likely resulted or contributed significantly to morbidity and death of these animals. These results expand the known geographical range of occurrence of crassicaudiasis in CBW. Specifically, the present study provides the first accounts of arterial and renal crassicaudiasis in CBW off the southern hemisphere, specifically in CBW off Brazil, and to the authors' knowledge, it is the first record of *C. anthonyi* in the southern Atlantic Ocean.

## Highlights

• First record of *Crassicauda anthonyi* in cetaceans off the southern Atlantic Ocean.
• Arterial and renal crassicaudiasis may be fatal in *Ziphius cavirostris*.
• ITS-2 gene sequence analysis enables *Crassicauda* species identification.

## Graphical abstract

**Figure:**
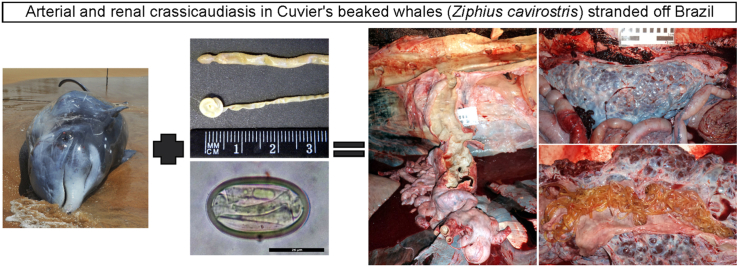

## 1 Introduction

Beaked whales (family Ziphiidae) encompass 22 current species (Mammalogy, 2015), of which nine are known to occur in Brazilian waters (Santos, 2016). The Cuvier's beaked whale (CBW; *Ziphius cavirostris*) is a cosmopolitan yet cryptic marine mammal found in deep tropical and temperate waters of all oceans (Baird, 2018). CBW is regarded as of least concern by IUCN Red List of Threatened Species. Knowledge on main threats to CBW is comparatively limited, however, anthropogenic (e.g., interaction with sonar [Fernandez et al., 2005], bycatch [Allen et al., 2012]), and infectious (e.g., herpesvirus [Arbelo et al., 2010; Felipe-Jiménez et al., 2021], morbillivirus [Centelleghe et al., 2017]) and non-infectious (e.g., Arbelo et al., 2013; Díaz-Delgado et al., 2018) natural causes of disease have been recorded. CBW's diet may vary according to geographic location although the most common items of prey include squids and teleost fish (West et al., 2017). CBW strandings have been reported sporadically along the Brazilian shore since 1969 albeit their distribution and population dynamics remain largely unknown in Brazilian waters (Carvalho, 1969; Mayorga et al., 2010; Bortolotto et al., 2016). Similarly, studies on diet of CBW's off Brazil are essentially lacking; the only information available refers to a pregnant CBW that had a piece of fishing net in the gastroesophageal segment, and a small amount of cephalopod beaks and fish otoliths (Bortolotto et al., 2016). Furthermore, there is very limited information regarding health status and causes of strandings and/or death for this species in Brazilian waters (Bortolotto et al., 2016).

Parasitic diseases are common in free-ranging cetaceans (Stroud and Roffe, 1979; Geraci and Aubin, 1987). *Crassicauda* genus (family Crassicaudidae; Order Spirurida) encompasses 14 species (*C. anthonyi, C. bennetti, C. boopis, C. crassicauda, C. fuelleborni, C. giliakiana, C. grampicola, C. magna, C. pacifica, C. tortilis, C. delamureana, C. pacifica*, *C. carbonelli*, and *C. duguyi*), based on morphologic features (Jabbar et al., 2015a). *Crassicauda* nematodes can affect several cetacean species and may be associated with severe, often fatal, gastrointestinal, respiratory, vascular, auditory and genitourinary lesions (Lambertsen, 1992; Jabbar et al., 2015a, Jabbar et al., 2015b; Díaz-Delgado et al., 2016). The ecology and life cycle of *Crassicauda* are not fully elucidated. An indirect and a direct life cycles have been postulated. In the former, which could occur at any host's age, *Crassicauda* larvae would be ingested through prey items (intermediate or paratenic hosts). By contrast, the direct transmission would ensue in lactating calves through ingestion of larvated eggs in urine-contaminated milk (Lambertsen, 1985, Lambertsen, 1992).

Records of *Crassicauda* in cetaceans off Brazil include *C. crassicauda* in penis, urethra and intestine in sei whale (*Balaenoptera borealis*) and fin whale (*B. physalus*) (Muniz-Pereira et al., 1999), and *Crassicauda* sp. in diaphragmatic pleura, suprascapular muscles, and penis in pygmy sperm whale (*Kogia breviceps*) (Carvalho et al., 2010). Nonetheless, lesions and/or deleterious effects were not documented in these accounts. Pterygoid osteolytic lesions presumptively associated with crassicaudiasis were described in Guiana dolphin (*Sotalia guianensis*) (Van Bressem et al., 2007) and common bottlenose dolphin (*Tursiops truncatus*) (Basiolio, 2017; Nepote, 2020). *Crassicauda anthonyi* was originally described based on specimens collected from the kidneys of the True's beaked whale (*Mesoplodon mirus*) off the Atlantic coast of France (Chabaud, 1962), and later reported in CBW from the Canary Islands (Manfredi et al., 2005), Costa Rica (Oliveira et al., 2011), Australia (Robson, 1984) and Puerto Rico (Mignucci-Giannoni et al., 1998), and fin whales off the Mediterranean Sea (Marcer et al., 2019); some of these records documented renal lesions including necrosis, fibrosis and partial destruction of the organ (Manfredi et al., 2005; Oliveira et al., 2011; Robson, 1984; Mignucci-Giannoni et al., 1998). There is lack of knowledge of *C. anthonyi* specific life history traits, transmission pathways and epidemiology.

Herein, we report the epidemiologic, pathologic, morphologic parasitologic features and molecular identification of arterial and renal crassicaudiasis by *Crassicauda* sp. in three geographically and chronologically distant CBW stranded off Brazil.

## 2 Materials and methods

### 2.1 Epidemiology and biologic data of investigated animals

CBW-1 was a 5.8 m-long, adult male in poor body condition and in moderate autolysis that was found stranded dead on December 21st, 2011, in Cidreira beach, Rio Grande do Sul State (30°09′23” S, 50°14′1” W). CBW-2 was a 6.0 m-long, adult female in moderate body condition that stranded alive on June 13th, 2012, in Pirambu (10°49′48.0” S; 36°55′55.9” W), Sergipe State. The animal died shortly after veterinary attention was initiated. CBW-3 was a 3,000 kg, 5.3 m-long, adult male in moderate body condition and mild to moderate autolysis that stranded dead in Guardo do Embaú beach, Santa Catarina State (27°56′24” S, 48°37′6” W), on February 14th, 2020, by The Santos Basin Beach Monitoring Project. These biologic and epidemiologic data are summarized in Table 1.

**Table 1:** Biologic and epidemiologic data of Cuvier's beaked whales (*Ziphius cavirostris*) off Brazil with crassicaudiasis included in this study.

Case	Sex	Age	Weight (kg)	Total body length (m)	Decomposition status	Body condition	Stranding date (mm/dd/yy)	Location	Coordinates
1	Male	Adult	NR	5.8	Moderate	Poor	12/21/2011	Cidreira (RS)	30°09′23”S, 50°14′1”W
2^a^	Female	Adult	NR	6	Fresh	Moderate	06/13/2012	Pirambu (SE)	10°49′48.0”S; 36°55′55.9”W
3	Male	Adult	3,000	5.3	Moderate	Moderate	02/14/2020	Guardo do Embaú (SC)	27°56′24”S, 48°37′6”W

a Stranded alive (visual confirmation). NR, not recorded; RS, Rio Grande do Sul State; SE, Sergipe State.

### 2.2 Postmortem pathologic examinations

Complete standardized necropsies were performed in the field for the three CBW (Geraci and Lounsbury, 2005; IBAMA, 2005). Representative tissue samples of main organs (skin, skeletal muscle, diaphragm, central nervous system, eye, pterygoid sac, tympanoperiotic complexes, tongue, oral mucosa, esophagus, stomach, small and large intestine, liver, pancreas, trachea, lung, heart, great vessels, kidney, ureter, urinary bladder, lymph nodes, spleen, testicle, penis, ovary, uterus, vagina, and vulva) were collected, fixed in 10% neutral buffered formalin, processed routinely through graded alcohols and embedded in paraffin-wax, and stained with hematoxylin and eosin (H&E) for histologic examination. Selected kidney and lung sections were stained with Masson's trichrome (for collagen) and Twort's Gram stain (for bacteria) to better characterize the lesions.

### 2.3 Parasitologic analyses

For morphologic parasitologic analysis, approximately 50 adult nematodes from kidney of each CBW were collected, preserved in 70% alcohol, cleared with lactophenol solution, and analyzed under an optical microscope. Nematode identification was based on taxonomic keys proposed by (Lambertsen, 1985; Anderson et al., 2009). Urine of CBW-2 was collected and analyzed under an optical microscope at 10 × magnification; no urine was readily available for collection and analysis in the other two individuals. For molecular analysis, we extracted DNA of two individual *Crassicauda* sp. preserved in alcohol from both CBW-2 and CBW-3 using DNeasy® Blood and Tissue Kit (Qiagen, Germany) according to the manufacturer's instructions. Genomic DNA extraction from formalin-fixed and paraffin-embedded sections of CBW-1 tissue was performed using QIAamp DNA FFPE Tissue (Qiagen, CA, USA) according to the manufacturer's recommendations. DNA extracts were amplified for the partial 18S and second internal transcribed spacer (ITS-2) region of the nuclear ribosomal DNA (rDNA). Polymerase chain reaction (PCR) was performed in 25 μL reactions containing 0.25 μM of each primer, 1x GoTaq® Green Master Mix (Promega Corporation, Madison, Wisconsin, United States) and 2.5 μL of DNA template. We amplified the 18S rDNA using the forward primer G18S4 (5′-GCTTGTCTCAAAGATTAAGCC-3′) and reverse 136 (5′-TGATCCTTCTGCAGGTTCACCTAC-3′) based on previously published sequences (Nadler et al., 2007). Amplification conditions consisted of an initial 95 °C for 2 min, followed by 35 cycles of 95 °C for 45 s, 51 °C for 45 s, and 72 °C for 1 min, and a final extension at 72 °C for 5 min. The ITS-2 region was amplified using the forward primer D (5′-GAGTCGATGAAGAACGCAG-3′) and reverse B1 (5′-GAATCCTGGTTAGTTTCTTTTCCT-3′) (Traversa et al., 2004). The cycling conditions consisted of an initial denaturation 95 °C for 2 min, followed by 35 cycles of 95 °C for 45 s, 50 °C for 45 s, and 72 °C for 45 s, and a final extension at 72 °C for 5 min. PCR products were purified using E.Z.N.A.® Cycle Pure Kit (Omega Bio-tek, USA), then sequenced in both directions using the original PCR primers in a 3730xl DNA Analyzer at Eurofins Genomics (Louisville, KY, United States). Phylogenetic analysis was inferred from the partial ITS-2 gene sequence using the Maximum Likelihood method with 1,000 bootstrap replicates in MEGA X 10.1 (Kumar et al., 2018). The best fit nucleotide substitution model for the data set was Hasegawa-Kishino-Yano (HKY). To guarantee phylogenetic tree reliability, a member of the same superfamily, *Habronema muscae*, was included as outgroup. Nucleotide sequences for the partial ITS-2 adult female and male (MZ363789 and MZ363790, respectively) and 18S of the same female and male (MZ362866 and MZ362867, respectively) regions have been deposited in GenBank.

### 2.4 Immunohistochemical analysis

Immunohistochemical (IHC) analysis for cetacean morbillivirus (CeMV) was performed in lung tissue sections of CBW-1 an employed a monoclonal antibody against the nucleoprotein of canine distemper virus (1:400 dilution; VMRD, Inc. Pullman, WA, USA), known to cross-react with CeMV. The streptavidin-biotin linked to alkaline phosphatase method was applied (DakoCytomation, CA, USA). Antigenic retrieval was achieved with citrate buffer pH 6.0 for 3 min in a microwave oven. Permanent Red (DakoCytomation, CA, USA) was used as chromogen. A case of canine distemper in a dog was used as positive control in this analysis, while the negative control consisted of replacing the primary antibody by phosphate buffered saline.

## 3 Results

### 3.1 Pathologic and immunohistochemical findings

**Fig. 1:**
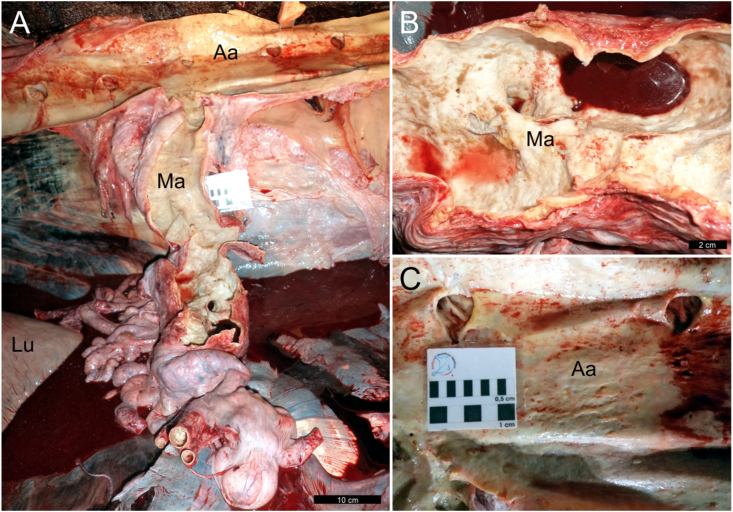
Macroscopic features of arterial and renal crassicaudiasis in Cuvier's beaked whales (CBW; *Ziphius cavirostris*). (**1**) CBW-2. The mesenteric arteries (A, B) and caudal abdominal aorta (C) are hard, non-collapsible, tortuous, and dilated. Aa, abdominal aorta; Ma, mesenteric artery; Lu, lung.

**Fig. 2:**
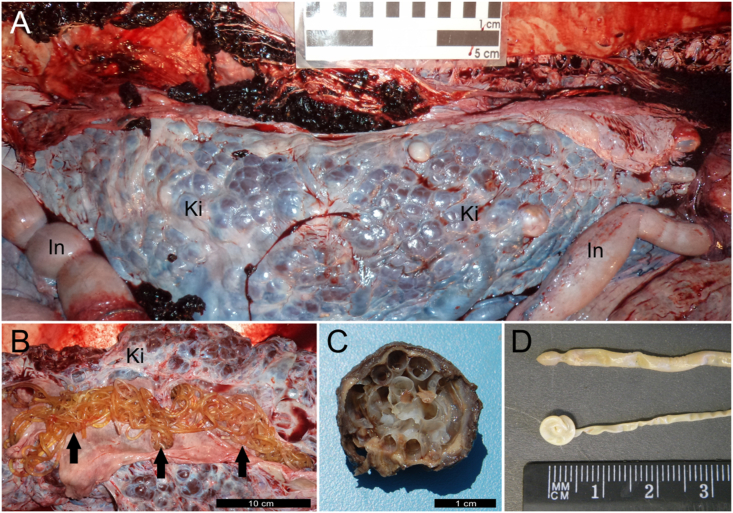
The kidneys are heavily infected (A [CBW-2], B [CBW-1]) by male and female adult *Crassicauda* sp., which penetrate the wall of the renicular calyces, ureters, and adjacent vasculature. (C) Renal granuloma (*ex situ*) with coiled (sectioned) adult *Crassicauda* nematode segments. (D) Posterior extremity of female (upper specimen) and male (lower specimen; characteristic coiled tail).

The main gross pathologic findings in CBW-1, CBW-2 and CBW-3 were confined to the cardiovascular and urinary systems (Table 2). The mesenteric arteries, caudal abdominal aorta, and renal arteries were hard, non-collapsible, tortuous, and dilated, up to 2 times the normal caliber of the distal and proximal branches (Fig. 1). Upon dissection, the arterial wall was thickened, up to 2 cm in the abdominal aorta. The kidneys were heavily infected by male and female adult *Crassicauda* sp., which penetrated the wall of the renicular calyces, ureters, and adjacent vasculature (Fig. 2). The nematodes extended into the interrenicular stroma where they were walled off by dense, fibrous connective tissue capsules and formed granulomas up to 3 cm diameter. The adjacent affected renal parenchyma exhibited areas of necrosis intercalated with firm, fibrous foci; renicular atrophy and hypertrophy were common within affected renal tissue. Some proximal ureteral and renicular pelvic spaces were dilated and filled with urine. Nematodes often obliterated the medium and small caliber renal arteries and veins. Additional gross findings in these animals were few non-healed to numerous, variably healed cookiecutter-shark (*Isistius brasiliensis*) bite wounds in CBW-2 and CBW-3. CBW-2 and CBW-3 had small numbers of subcutaneous *Phyllobothrium delphini* metacestodes (up to 7 mm in diameter) (Agusti et al., 2005), more prominent in the caudal abdominal and perigenital regions. CBW-2 had multifocal ulcerative gastritis with intralesional *Anisakis* sp. nematodes, as well as pulmonary edema and presumptive costal osteoporosis.

**Table 2:** Pathologic and parasitologic findings in Cuvier's beaked whales (*Ziphius cavirostris*) off Brazil included in this study.

Case	Pathology: Etiological diagnoses	Parasitology
1	Arterial (mesenteric, caudal abdominal aorta, renal) and renal crassicaudiasis; Bacterial bronchopneumonia (Gram-negative bacilli); Thromboembolic hepatitis (presumptively bacterial origin); Cutaneous cookiecutter-shark (*Isistius brasiliensis*) bite wounds	Morphologic analysis (adults): *Crassicauda* sp.
2^a^	Arterial (mesenteric, caudal abdominal aorta, renal) and renal crassicaudiasis; Cutaneous cookiecutter-shark (*Isistius brasiliensis*) bite wounds; Hepatic steatosis with congestion and hemorrhage; Pulmonary emphysema, edema, and hemorrhage; Gastric anisakiasis	Morphologic analysis (adults): *Crassicauda* sp.; *Anisakis* sp. Molecular analysis: *C. anthonyi*.
3	Arterial (mesenteric, caudal abdominal aorta, and renal) and renal crassicaudiasis; Cutaneous cookiecutter-shark (*Isistius brasiliensis*) bite wounds; Cutaneous cestodiasis (*Phyllobothrium delphini*)	Morphologic analysis (adults): *Crassicauda* sp., *Phyllobothrium delphini* Molecular analysis: *C. anthonyi*.

a Stranded alive (visual confirmation). NR, not recorded; RS, Rio Grande do Sul State; SE, Sergipe State.

Microscopically, in all three animals, grossly affected arterial segments exhibited severe, chronic proliferative and fibrosing endarteritis with multifocal mineralization and cartilaginous and osseous metaplasia. Pleocellular inflammatory infiltrates, including neutrophils, eosinophils, macrophages, and lymphocytes, as well as hemorrhage and fibrin were seen in the arterial intima in earlier lesions. In the kidneys, lesions observed varied between areas affected and included pyogranulomatous pyelonephritis with nematodes within the calices and dilated pelvices; coagulative necrosis adjacent to areas of interstitial fibrosis with atrophy and extensive loss of nephrons; arteritis and phlebitis; and chronic suppurative and sclerosing ureteritis with intralesional nematodes. Adult *Crassicauda* nematodes had thin and smooth cuticle, coelomyrian musculature, large paired multinucleated lateral chords, pseudocoelomic cavity, uterus containing numerous round to oval thick-shelled larvated eggs (up to 19.76 × 31.76 μm) and intestine composed of cuboidal to columnar cells with brush borders. Additional findings in CBW-1 were marked suppurative bronchopneumonia with fibrinocellular thrombosis and intralesional gram-negative bacilli (Supplemental Fig. 1), and thromboembolic necrotizing hepatitis. CBW-2 had moderate to marked, multifocal, acute centrilobular hepatocellular vacuolar (lipid type) degeneration with congestion and hemorrhage, and pulmonary emphysema, edema, and hemorrhage. IHC analysis for CeMV in lung tissue of CBW-1 was negative.

### 3.2 Parasitologic findings

**Fig. 3:**
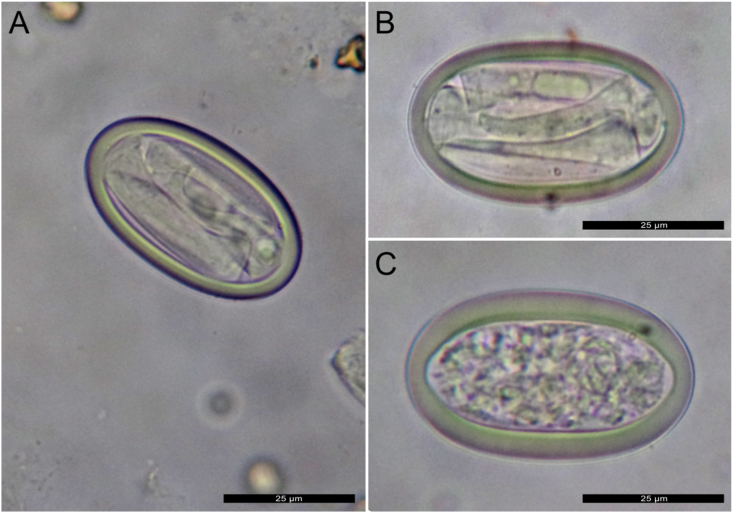
Typical *Crassicauda* oval-shaped larvated (A, B) and embryonated (C) eggs in urine of CBW-2.

Parasitologic analyses in urine of CBW-2 identified typical *Crassicauda* oval-shaped and larvated eggs (Fig. 3). Adult nematodes removed from the three CBW were identified as *Crassicauda* sp. based on morphologic and morphometric characteristics of the species’ posterior end. Females were 100–170 cm-long, 4 mm-width and had a posterior narrowing. Males were 27–34 cm-long, 2 mm-width and exhibited a corkscrew-shaped posterior end.

**Fig. 4:**
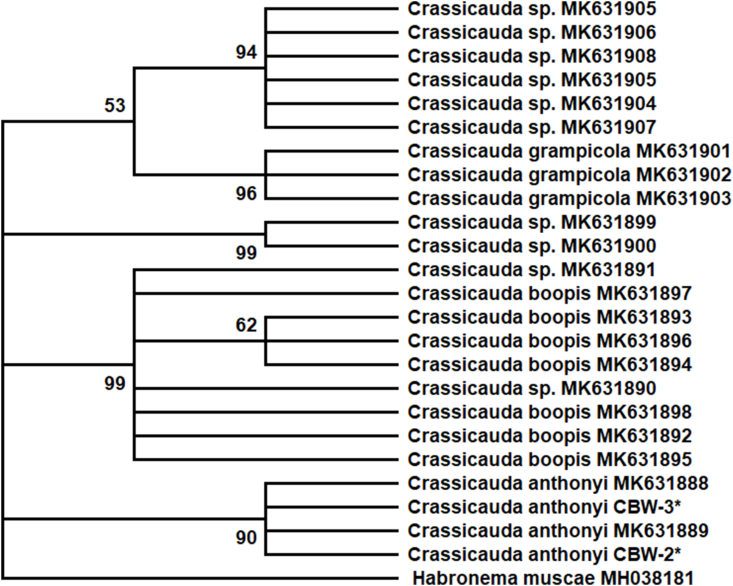
Phylogenetic analysis. Molecular analyses identified the specimens collected from CBW2 and CBW3 as *Crassicauda anthonyi,* based on the ITS2 region, and supported by phylogenetic analysis. Analysis was performed by MEGA X 10.1 using the maximum likelihood method (1,000 bootstrap replicates) and included *Habronema muscae* as outgroup. GenBank accession numbers are listed along the species names. Branches with bootstrap support lower than 50% were collapsed. *Sequences obtained in this study.

**Fig. 5:**
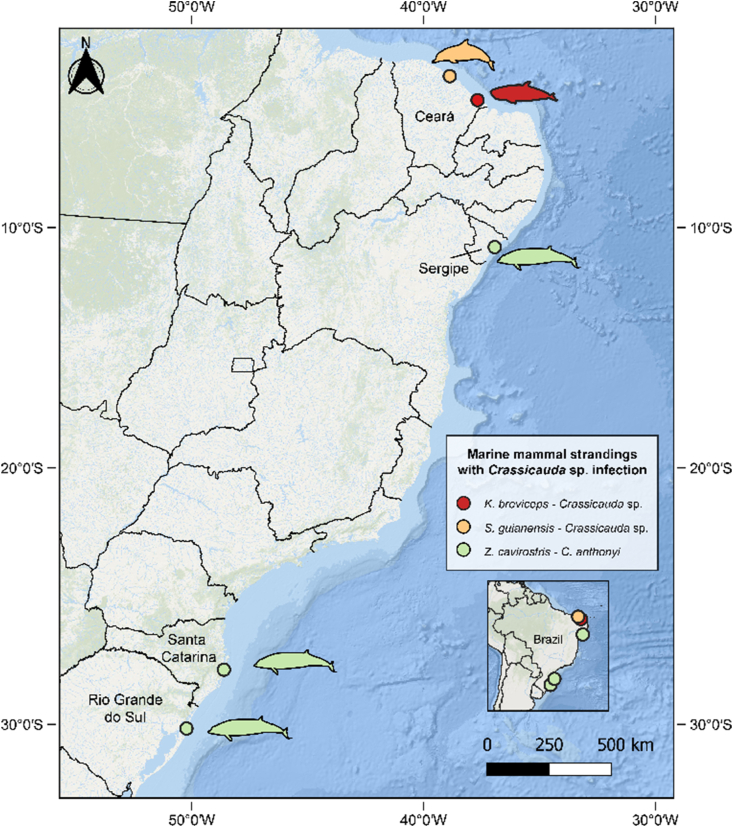
Records of Crassicauda sp. in cetaceans stranded along the coast of Brazil including the three Cuvier beaked whales (Ziphius cavirostris) from this study, a pygmy sperm whale (Kogia breviceps) (Carvalho et al., 2010), and a Guiana dolphin (Sotalia guianensis) (Carvalho V.L., personal communication).

Molecular analyses identified the specimens collected from CBW2 and CBW3 as *Crassicauda anthonyi,* based on the ITS2 region, and supported by phylogenetic analysis (Fig. 4). The BLAST analysis demonstrated that CBW-2 and CBW-3 sequences presented a 100% nucleotide homology with sequences of C. anthonyi available in GenBank. The ITS2 fragments amplified were 549 base pairs (bp) and clustered with *C. anthonyi* from Italy (MK631888 and MK631889) with 90% bootstrap support. We characterized for the first time the 18S region of *C. anthonyi* (1,690 bp). The 18S region revealed to be highly similar between *Crassicauda* species available in GenBank, therefore, phylogenetic analysis was not performed on these fragments. PCR was not successful for the formalin-fixed material of CBW-1. A map illustrating stranding locations on the Brazilian coast and *Crassicauda* sp. identified in cetaceans off Brazilian waters is provided in Fig. 5.

## 4 Discussion

Current medico-pathologic knowledge of CBW remains limited if compared to growing knowledge in other cetacean species. Known causes of typical or atypical mass strandings of CBW have involved barotrauma from naval mid-frequency active sonar and gas and fat embolic syndrome (Fernandez et al., 2005; Bernaldo de Quiros et al., 2019). CBW single strandings and/or fatalities have been associated with viral e.g., dolphin morbillivirus (Centelleghe et al., 2017), herpesvirus (Arbelo et al., 2010; Felipe-Jiménez et al., 2021), bacterial e.g., *Citrobacter freundii* (Fernández et al., 2011), and parasitic infections by *C. anthonyi* (Mignucci-Giannoni et al., 1998; Oliveira et al., 2011) and *C. magna* (Díaz-Delgado et al., 2016), as well as gastrointestinal foreign bodies, vessel collision, and interaction with fishing activities (Díaz-Delgado et al., 2018; Puig-Lozano et al., 2018, 2020).

The three CBW in this study had similar *Crassicauda*-associated pathologic findings among them, namely severe, chronic proliferative mesenteric and caudal aortic endarteritis and chronic granulomatous and fibrosing interstitial nephritis with renicular atrophy and loss, and numerous intralesional *Crassicauda* nematodes, which in CBW2 and CBW3 were identified as *C. anthonyi*. This is in agreement with previous observations in CBW and supports a direct association between arteritis and parasitism by *Crassicauda* sp. (Díaz-Delgado et al., 2016). Severe arterial and renal crassicaudiasis is a significant cause of morbidity and mortality in these animals. Comparatively, venous lesions, primarily in vena cava, and subsequent pulmonary emboli, as observed in blue whale (*Balaenoptera musculus*), fin whale and humpback whales (*Megaptera novaeangliae*) with crassicaudiasis (Lambertsen, 1992), have not been documented in CBW. The origin of pulmonary and hepatic thromboemboli in CBW-1 is believed to be the result of lung disease and subsequent vascular involvement, and likely contributed to multiorgan compromise. The differences aforementioned may have transmission implications because in CBW as well as in large whales, urinary excretion of *Crassicauda* eggs is expected; *C. anthonyi* eggs were morphologically confirmed in urine of CBW-2. A second hypothetical transmission route in fin whales with embolized pulmonary eggs would involve expelling through exhaled air (Cockrill, 1960). Nonetheless, such a route remains to be demonstrated in fin whales and is highly unlikely to occur in CBW given the lack of embolized eggs in lung by histopathologic examinations.

Additional pathologic findings in CBW-1 included a severe suppurative bronchopneumonia with intralesional gram-negative bacilli, which may have occurred as a result of aspiration of regurgitated material; a primary bacterial respiratory infection could not be excluded. Bacterial culture was not performed in affected lung tissue; thus, no further conclusions can be drawn concerning the bacterial agent(s) involved. Immunohistochemical analysis for CeMV, which was recently detected for the first time in CBW (Centelleghe et al., 2017), was negative in the present case.

*Crassicauda anthonyi* was originally described based on specimens collected from the kidneys of the True's beaked whale (*Mesoplodon mirus*) off the Atlantic coast of France (Chabaud, 1962), and later reported in CBW from the Canary Islands (Manfredi et al., 2005), Costa Rica (Oliveira et al., 2011), Australia (Robson, 1984) and Puerto Rico (Mignucci-Giannoni et al., 1998), and fin whales off the Mediterranean Sea (Marcer et al., 2019). To the best of the authors knowledge, this is the first report of *C. anthonyi* from the southern Atlantic Ocean.

Similar to previous studies, we conclude that the 18S region (1,690 bp) is highly conserved among *Crassicauda* species, and while it may be useful to confirm genus-level identification, it is not an informative marker for *Crassicauda* species-level identification (Jabbar et al., 2015a, Jabbar et al., 2015b; Marcer et al., 2019). Hence, no species identification at the 18S region was pursued in our cases. In contrast, the ITS-2 region supported the identity of our isolates as *C. anthonyi* available at GenBank and has proved to be an efficient DNA barcode marker for molecular Crassicauda species identification as demonstrated by Marcer et al. (2019). Future studies are warranted to further elucidate epidemiologic features of crassicaudiasis in CBW in Brazil. Establishment of and cooperation among stranding networks with emphasis on coastal areas understudied coupled with greater analytic effort, including diet composition analyses, will enable greater understanding of this condition.

In conclusion, severe arterial and renal crassicaudiasis due to *C. anthonyi* in these CBW likely resulted or contributed significantly to morbidity and death. These results expand the known geographical range of occurrence of crassicaudiasis in CBW. Specifically, the present study provides the first accounts of arterial and renal crassicaudiasis in CBW off the southern hemisphere, specifically in CBW of Brazil, and appears to be the first record of *C. anthonyi* in the southern Atlantic Ocean.

## Declaration of conflicting interests

The authors declare no potential conflicts of interest with respect to the research, authorship and/or publication of this article.

## Appendix A Supplementary data

figs1 figs1
