# Supplementary figures and images for: Crassicaudiasis in three geographically and chronologically distant Cuvier's beaked whales (Ziphius cavirostris) stranded off Brazil
Source: Int J Parasitol Parasites Wildl. 2021 Nov 6;16:262–9. doi: 10.1016/j.ijppaw.2021.10.010 (PMC8605309; doi:10.1016/j.ijppaw.2021.10.010)

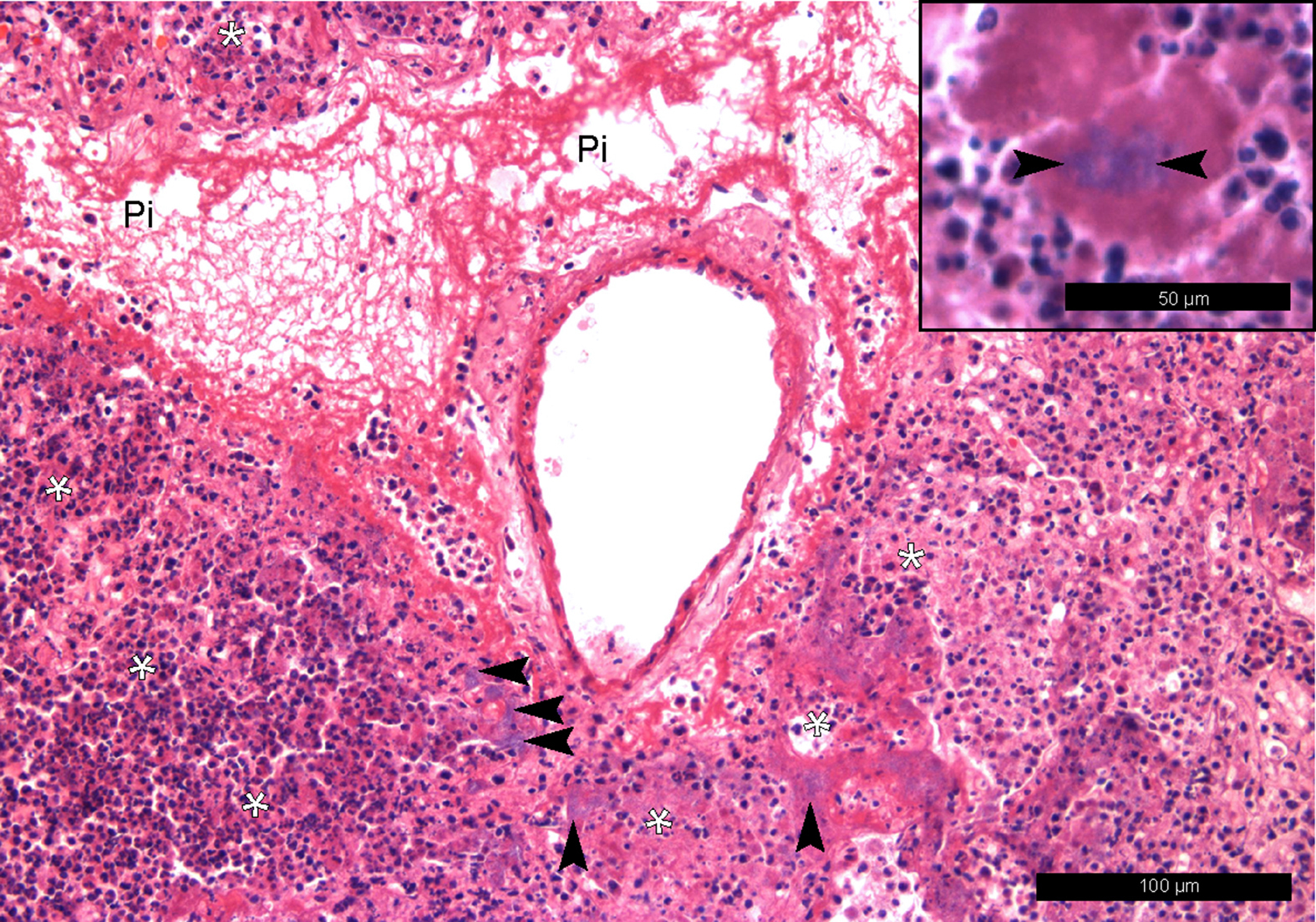

Supplement: figs1 [file mmcfigs1.jpg]
